# A Policy Support Analysis of Air Pollution Epidemiology and Hospital Admissions in Kuwait

**DOI:** 10.1007/s44197-026-00609-9

**Published:** 2026-07-09

**Authors:** Yazan Alwadi, Shereef S. Al-Khayat, Maryam A. Al-Hunaidi, Hanan S. Mallallah, Nawaf S. Alhajeri, Jing Li, Ali Al-Hemoud, Petros Koutrakis, Barrak Alahmad

**Affiliations:** 1 Environmental Health Department, Harvard T.H. Chan School of Public Health, Harvard University, Boston, MA USA; 2 Air Pollution Monitoring Department, Kuwait Environment Public Authority (EPA), Kuwait City, Kuwait; 3 Department of Environmental Sciences, College of Life Sciences, Kuwait University, Kuwait, Kuwait; 4 Institute of Child and Adolescent Health, School of Public Health, Peking University, Beijing, China; 5 Environment and Life Sciences Research Center, Kuwait Institute for Scientific Research (KISR), Safat, Kuwait; 6 Dasman Diabetes Institute, Kuwait City, Kuwait; 7 Directorate of Public Health, Ministry of Health, Safat, Kuwait

**Keywords:** PM2.5, Air pollution, Hospital admissions, Cardiopulmonary diseases, Environmental epidemiology, Air quality policy, Attributable burden, Population-weighted exposure, Kuwait, Gulf Cooperation Council (GCC)

## Abstract

**Background:**

Gulf Cooperation Council (GCC) countries have long lacked local epidemiological evidence demonstrating the health benefits of reducing air pollution. In Kuwait, however, the past decade has brought new, detailed health and environmental exposure data that now make such assessments possible.

**Objective:**

To present an epidemiological basis in support for a strict air quality policy in Kuwait.

**Methods:**

We collected all cardiopulmonary hospital admissions from six public hospitals in the country from 2010 to 2018. We then matched cases with daily population-weighted PM_2.5_ exposure estimated at 1 km² resolution. For each hospital service region, we fitted generalized additive models with a negative binomial distribution that estimated the relative risk of admission per 10 µg/m³ increase in PM_2.5_. We then calculated attributable admissions under three hypothetical policy scenarios: (1) maintaining the status quo (‘do nothing’), (2) reducing PM_2.5_ concentrations by 10 µg/m³, and (3) reducing by 20 µg/m³.

**Results:**

There was a total of 380,862 cardiopulmonary hospital admissions during the study period. Every 10 µg/m³ increase in PM_2.5_ was associated with a 1% (95%CI: 0.6%-1.5%) increase in cardiopulmonary admission. Under the status quo, 3.4–3.9% of cases can be attributed to PM_2.5_, totaling 1,536 (95%CI: 956-2,108) excess admissions/year. A 10 µg/m³ reduction could prevent up to 403 (95%CI: 252–551) admissions/year; a 20 µg/m³ reduction up to 810 (95%CI: 505-1,109) admissions/year.

**Conclusion:**

Reducing air pollution, even modestly, offers measurable public health benefits. Data from Kuwait provide a model for evidence-based policymaking and can help guide other GCC countries with similar pollution profiles toward adopting stricter air quality standards aligned with WHO interim targets.

## Introduction

Inhalation of fine particulate matter (particles with an aerodynamic diameter less than 2.5 microns; PM_2.5_) has been linked to a number of harmful health impacts [1, 2]. Due to their small size, these particles bypass primary physiological defense mechanisms and penetrate deep into the lungs and the bloodstream. Once absorbed, PM_2.5_ particles can trigger inflammation and oxidative stress [3]. Global scientific evidence consistently shows that high levels of PM_2.5_ exposure increase hospital admissions for heart and lung diseases [4–7]. Healthcare systems and hospitals can become overwhelmed as admissions rise.

In 2021, the World Health Organization (WHO) released updated and stricter air quality standards. Without strong local epidemiological evidence demonstrating health benefits, Gulf Cooperation Council (GCC) countries have struggled to meet the WHO interim targets. In Kuwait, the past decade has seen increased availability of detailed health and environmental exposure data, enabling such investigations. A growing body of evidence is now emerging on the adverse health effects of air pollution in Kuwait [8–12]. This creates a unique timely opportunity in the region to generate detailed epidemiological evidence that supports strict air quality policy action. Such analyses would quantify the burden air pollution places on local healthcare systems and identify communities at greatest risk.

The Kuwait Environmental Public Authority (KEPA) commissioned a collaborative research effort with the Harvard T.H. Chan School of Public Health and the Kuwait Institute for Scientific Research with a primary goal to mitigate exposure to air pollution across Kuwait. Policymakers asked for clear data linking existing pollution levels to tangible local health outcomes before updating national air quality standards. We were charged with two objectives: first, to quantify the overall health burden attributable to PM_2.5_ exposure in Kuwait; and second, to estimate the potential health benefits under various PM_2.5_ reduction scenarios.

To answer the two questions, we first collected hospital admissions data from all public hospitals in Kuwait. We then developed high-resolution PM_2.5_ exposure models that can cover the locations for the same study period. For each day and hospital service region, we calculated the average PM_2.5_ concentration. Using these data, we estimated the number of excess hospital admissions attributable to PM_2.5_ under three policy-relevant scenarios: [1] maintaining the status quo (‘do nothing’), [2] reducing PM_2.5_ concentrations by 10 µg/m³, and [3] reducing by 20 µg/m³. This study is the first of its kind in Kuwait and the Gulf region to integrate detailed local hospital data with advanced air pollution modeling techniques to systematically quantify the healthcare burden associated with fine particulate matter.

## Methods

### Hospital Data

Hospital admissions for cardiopulmonary diseases were obtained from the Kuwait Ministry of Health’s National Center for Health Information. The dataset included records of every admission from all public hospitals across Kuwait. We specifically identified cardiovascular and respiratory (cardiopulmonary) conditions using the World Health Organization, International Classification of Diseases, Tenth Revision (ICD-10), specifically codes I00-I99 for cardiovascular diseases (e.g., ischemic heart disease, heart failure, hypertension) and J00-J99 for respiratory diseases (e.g., asthma, chronic obstructive pulmonary disease, pneumonia). Public hospital data was available for every day between January 1, 2010, and December 31, 2018. We limited the analysis to six large public hospitals (Table 1). We excluded Jaber Al-Ahmad Hospital due to insufficient data availability following its opening in 2018. Population data corresponding to each hospital’s service area were obtained from Kuwait’s Central Statistical Bureau.

**Table 1:** Summary of cardiopulmonary hospital admissions in Kuwait’s public hospitals for the study period (2010–2018)

Hospital	Total Cases	% of admissions	Average Cases per day (standard deviation)
*Al-Adan*	77,131	20.3%	23.5 (7.7)
*Al-Amiri*	45,617	12.0%	13.9 (5)
*Al-Sabah*	49,010	12.9%	14.9 (10)
*Farwaniya*	82,169	21.6%	25 (7.3)
*Jahra*	64,908	17.0%	19.7 (7.6)
*Mubarak Al-Kabir*	62,027	16.3%	18.9 (5.5)
***Total***	**380**,**862**	**100.0%**	**115.9 (7.1)**

### Environmental Data

We used newly developed PM_2.5_ predictions for historical exposure, based on models developed by the Harvard T.H. Chan School of Public Health. The methods to derive exact concentrations are described in detail in Li et al. [13, 14]. Briefly, this high-resolution dataset provides daily mean PM2.5 concentrations at a spatial resolution of approximately every 1 km^2^ across Iraq and Kuwait. The predictions were generated using a combination of random forest machine learning algorithms and generalized additive mixed models to estimate daily visibility. Visibility estimates were derived from satellite-based aerosol optical depth (AOD) data and regional airport visibility records. These were then converted into PM₂.₅ concentrations using empirically derived relationships from regional ground monitoring stations. The resulting PM₂.₅ predictions were further refined using meteorological parameters, land-use features, vegetation indices, and satellite-derived surface dust as a proxy for sandstorm activity. We obtained these modeled daily PM₂.₅ gridded predictions and converted them into raster surfaces, which were spatially linked to hospital service area polygons for exposure assessment.

**Fig. 1:**
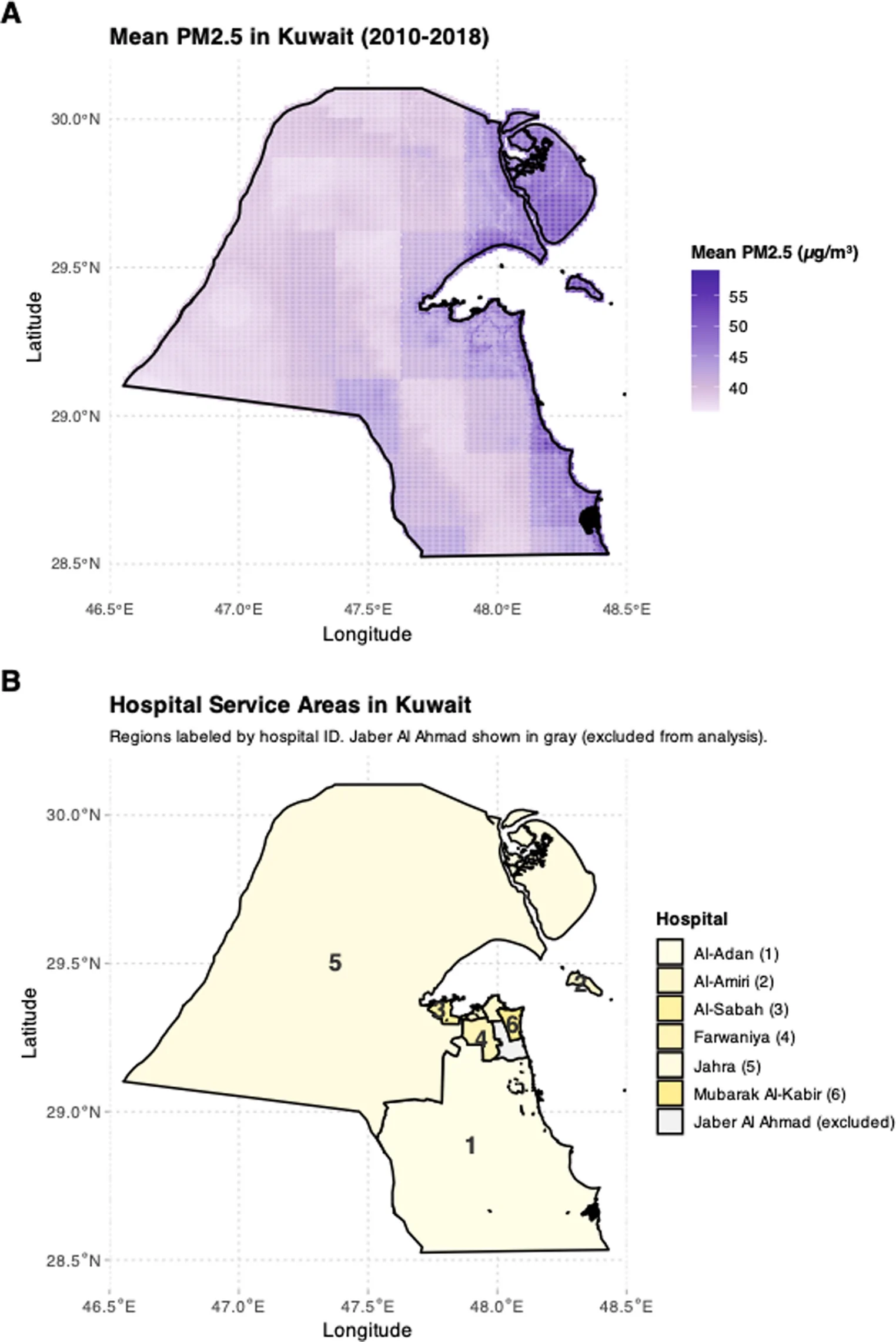
Mean PM2.5 (2010–2018) and public hospital service areas in Kuwait

Figure 1A shows the spatial distribution of mean PM₂.₅ concentrations across Kuwait averaged over the study period (2010–2018).

To more accurately reflect population exposure within each hospital service polygon, we obtained yearly population count data at a 1 km × 1 km resolution from publicly available sources [15]. Each grid cell’s daily PM_2.5_ concentration was weighted by the number of people residing within that cell. Grid cells partially intersecting hospital service area boundaries were weighted according to the proportion of the grid-cell area contained within each polygon. The population-weighted PM2.5 values were then summed and divided by the total population in each service area polygon, yielding daily population-weighted mean PM2.5 exposure levels for each hospital service area. Figure 1 bottom panel shows Kuwait’s Hospitals service areas.

We obtained daily mean temperature data for the same study period, at a spatial resolution of approximately 9 km from the ERA5-Land daily aggregated dataset provided by the Copernicus Climate Data Store (CDS). The ERA5-Land dataset provides globally consistent, high-quality daily climate estimates derived from satellite observations, in-situ measurements, and advanced atmospheric modeling techniques. Such satellite products are particularly suitable for epidemiological studies in regions with limited observational data. For each hospital service area polygon, daily mean temperature was estimated by averaging temperature values across all intersecting grid cells. Grid cells partially overlapping service area boundaries were weighted proportionally based on the fraction of their area falling within each respective hospital polygon.

### Statistical Analysis

We used generalized additive models (GAM) with a negative binomial distribution to estimate the association between daily PM2.5 exposure and daily hospital admissions for cardiopulmonary conditions. After checking for non-linearity, the final model included a linear term for PM2.5 exposure, fixed effects for each hospital service area, and adjustments for long-term trends, seasonality, and day of the week patterns. Long-term trends and seasonality were modeled using a natural spline with 4 degrees of freedom per year (total of 36 degrees of freedom for years of data included). Day-of-week effects were included as categorical variables (Sunday to Saturday).

To control for potential confounding by ambient temperature, we used a distributed lag nonlinear model (DLNM) with a maximum lag of 30 days. Specifically, we constructed a cross-basis function for daily mean temperature, using natural cubic splines with internal knots placed at the 10th, 75th, and 90th percentiles of the temperature distribution, and spline knots for lag structure were placed at logarithmically equally spaced intervals. We used DLNM model parameters that were previously tested and optimized for Kuwait [16].

To quantify the public health burden associated with PM_2.5_ exposure, we calculated the attributable fraction, representing the proportion of cardiopulmonary hospital admissions attributable to PM_2.5_ exposure. We selected a reference PM2.5 exposure level of 0 µg/m³ to estimate the overall impact of particulate matter pollution. The number of attributable cases was then obtained by multiplying the daily attributable fraction by the observed number of hospital admissions for each hospital service area.

## Results

We analyzed a total of 380,862 cardiopulmonary cases admitted to Kuwait hospitals during the entire study period. Farwaniya Hospital reported the highest number of cases, with 82,169 admissions, accounting for 21.6% of the national total. It also had the highest average daily case count (25 ± 7.3 cases/day). Al-Adan Hospital followed with 77,131 admissions (20.3% of total). Al-Amiri Hospital had the lowest number and daily cases (45,617 total; 13.9 ± 5 cases/day).

Table 2 presents descriptive statistics for the primary variables included in the analysis. Across all hospital service areas and study days, the mean daily PM₂.₅ concentration was 38.3 µg/m³ (SD: 14.6), while the mean daily temperature was 27.0 °C (SD: 9.3). Daily cardiopulmonary admissions averaged 18.2 cases (SD: 8.3).

**Table 2:** Summary statistics of daily cardiopulmonary admissions, PM₂.₅ concentrations, and mean temperature across all hospital service areas in Kuwait (2010–2018)

Variable	Minimum	Maximum	Mean	Median	SD
Cardiopulmonary admissions	1.0	55.0	18.2	17.0	8.3
PM2.5 (µg/m³)	19.3	200.1	38.3	36.0	14.6
Mean temperature (°C)	4.9	44.0	27.0	27.8	9.3

**Fig. 2:**
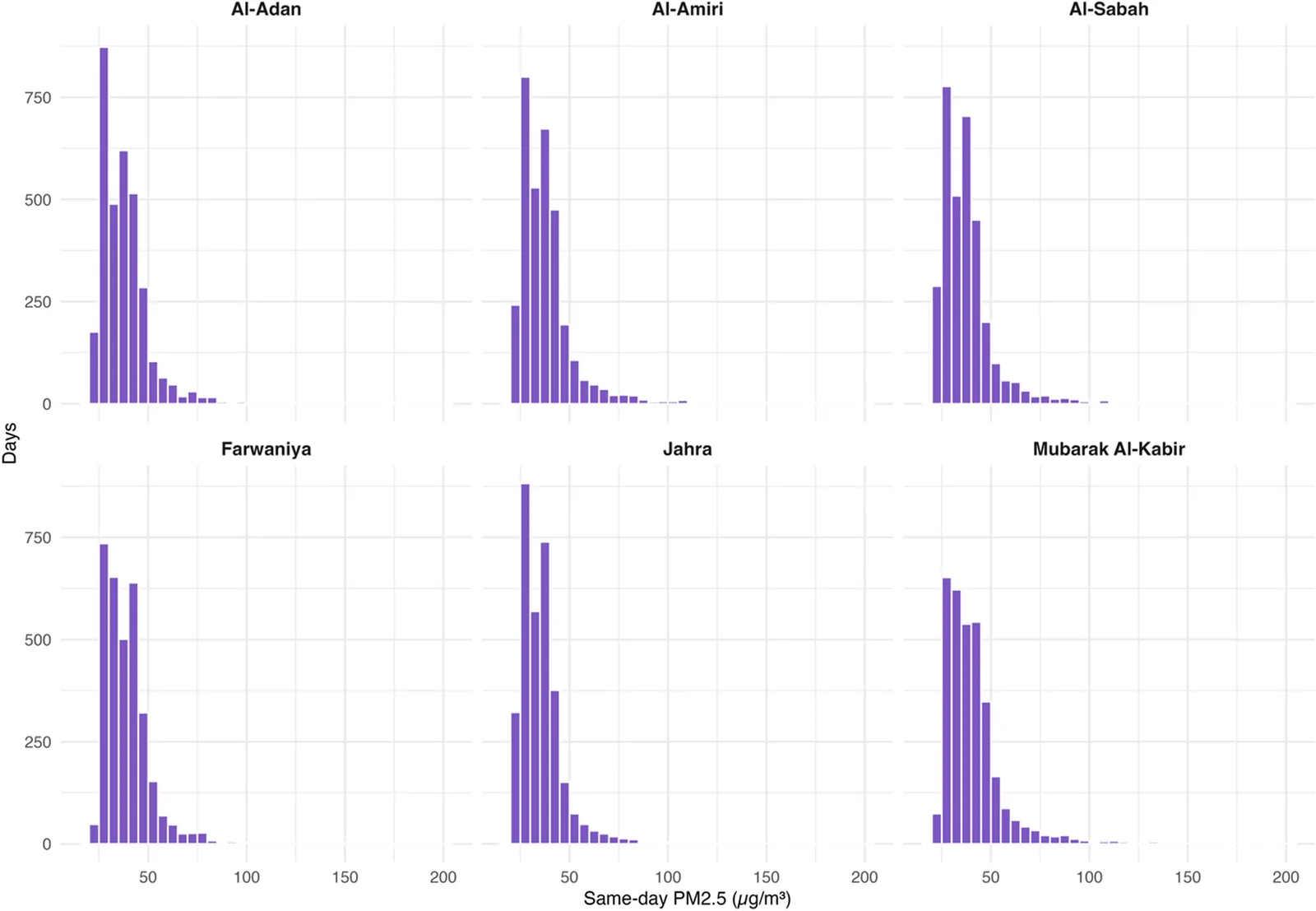
Distribution of same-day (lag 0) population-weighted PM₂.₅ concentrations by public hospital service area in Kuwait during the study period (2010–2018)

Overall, across the study period (2010–2018), average PM_2.5_ was 38.3 µg/m³ across the country. The distributions of daily population-weighted PM2.5 concentrations for each hospital service area in Kuwait is presented in Fig. 2. Across all hospitals, the distributions were right-skewed (episodic high-exposure days). Mubarak Al-Kabir had the highest mean daily PM_2.5_ concentration (41.0 µg/m³), followed by Farwaniya (39.3 µg/m³) and Al-Amiri (38.2 µg/m³). Jahra had the lowest average exposure (35.8 µg/m³). Median values ranged from 33.9 µg/m³ (Jahra) to 38.2 µg/m³ (Mubarak Al-Kabir). Maximum daily concentrations exceeded 180 µg/m³ in all hospitals. The highest air pollution episode reached around 200.1 µg/m³ in Mubarak Al-Kabir.

**Fig. 3:**
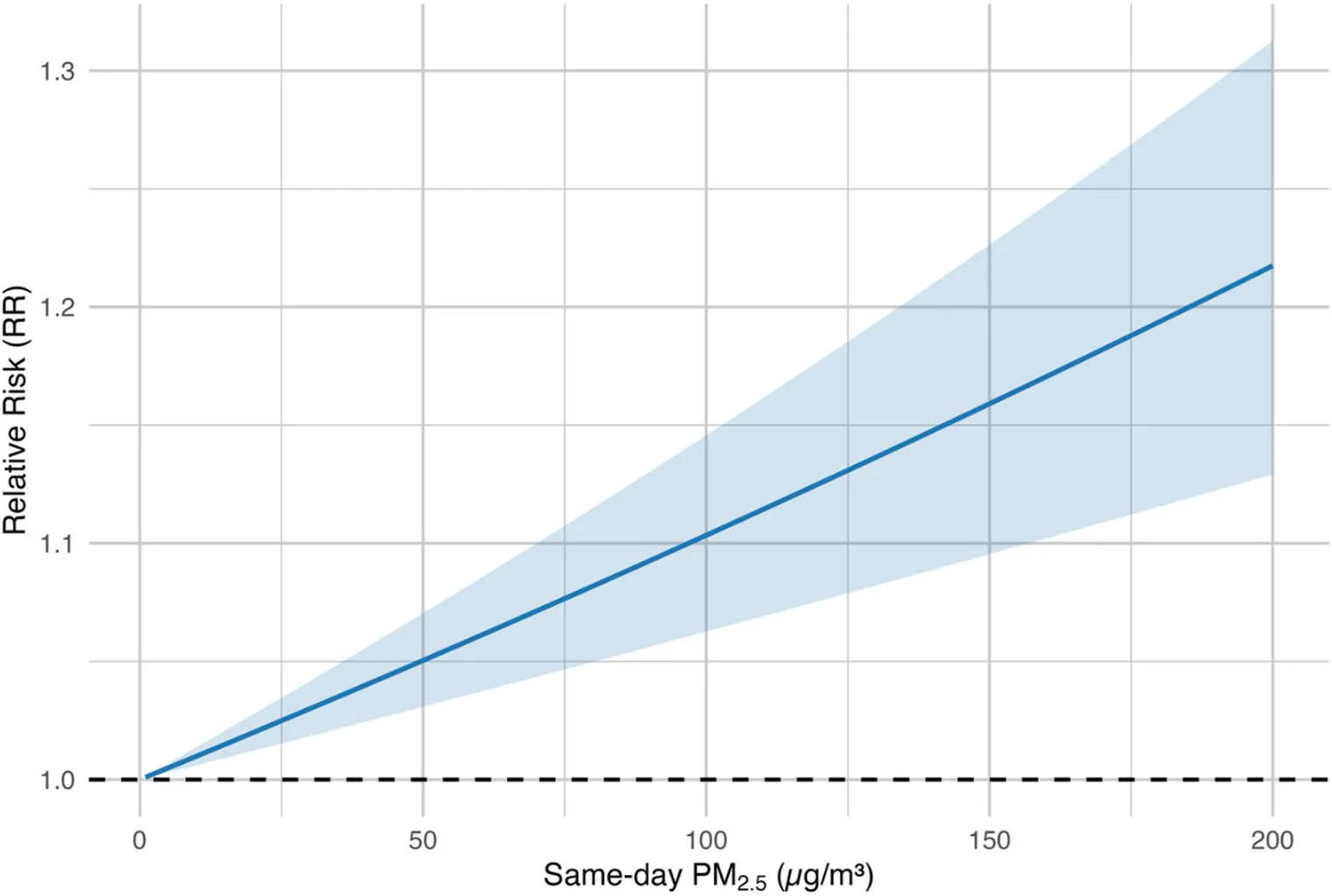
Estimated relative risk of cardiopulmonary admissions associated with PM_2.5_ exposure in Kuwait

The estimated relative risk (RR) of cardiopulmonary admissions associated with PM_2.5_ exposure resulted in a monotonic increase in risk with higher exposures (Fig. 3). For each 10 µg/m³ increase in PM_2.5_, the RR increased by approximately 1%. At 100 µg/m³, the RR was 1.10 (95% CI: 1.06–1.15), and at 150 µg/m³, it reached 1.16 (95% CI: 1.09–1.22), relative to a theoretical counterfactual concentration of 0 µg/m³. In other words, this translated to a 10% higher risk at 100 µg/m³ vs. days with hypothetically no PM_2.5_ exposure. Similarly, it was 16% higher in days with 150 µg/m³. Lagged exposures showed similar associations. Adjustments to temperature did not change the main results.

Our modeled three policy-relevant scenarios are shown in Table 3. In the first, continuing the status quo (Scenario 1), the proportion of cardiopulmonary hospital admissions attributable to fine particulate pollution ranged from 3.4% (95% CI: 2.1–4.6%) in Jahra to 3.9% (95% CI: 2.4–5.3%) in Mubarak Al-Kabir. This corresponded to an estimate from 1633 (95% CI: 1016–2239) total excess admissions over the study period in Al-Amiri and up to 3044 (95% CI: 1894–4174) in Farwaniya (the highest among all hospitals). When annualized, these values translated into 181 (95% CI: 113–249) and 338 (95% CI: 210–464) excess admissions per year, respectively. Overall, PM2.5 contributed to a total of 1,536 (95% CI: 956–2,108) excess admissions/year.

**Table 3:** Attributable burden of cardiopulmonary admissions under different simulated scenarios of PM_2.5_ exposure in Kuwait

Hospital	Attributable Fraction (95% CI)	Attributable Cases, overall (95% CI)		Attributable Cases, per year (95% CI)	Reduced Cases, per year (95% CI)
**Scenario 1**	‘Do Nothing’ Maintain Status Quo				
*Al-Adan*	3.6% (2.2–4.9%)	2751 (1712–3774)		306 (190–419)	0
*Al-Amiri*	3.6% (2.2–4.9%)	1633 (1016–2239)		181 (113–249)	0
*Al-Sabah*	3.7% (2.3–5.1%)	1817 (1132–2490)		202 (126–277)	0
*Farwaniya*	3.7% (2.3–5.1%)	3044 (1894–4174)		338 (210–464)	0
*Jahra*	3.4% (2.1–4.6%)	2188 (1361–3003)		243 (151–334)	0
*Mubarak Al-Kabir*	3.9% (2.4–5.3%)	2397 (1493–3285)		266 (166–365)	0
**Scenario 2**	Hypothetical scenario of reducing PM2.5 by 10 µg/m³				
*Al-Adan*	2.6% (1.6–3.6%)	2016 (1252–2769)		224 (139–308)	82 (51–111)
*Al-Amiri*	2.6% (1.6–3.6%)	1198 (744–1645)		133 (83–183)	48 (30–66)
*Al-Sabah*	2.8% (1.7–3.8%)	1350 (840–1853)		150 (93–206)	52 (33–71)
*Farwaniya*	2.8% (1.7–3.8%)	2261 (1405–3106)		251 (156–345)	87 (54–119)
*Jahra*	2.4% (1.5–3.3%)	1568 (974–2155)		174 (108–239)	69 (43–95)
*Mubarak Al-Kabir*	2.9% (1.8–4.0%)	1807 (1124–2481)		201 (125–276)	65 (41–89)
**Scenario 3**	Hypothetical scenario of reducing PM2.5 by 20 µg/m³				
*Al-Adan*	1.7% (1.0–2.3%)	1273 (790–1751)		141 (88–195)	165 (102–224)
*Al-Amiri*	1.7% (1.0–2.3%)	759 (471–1043)		84 (52–116)	97 (61–133)
*Al-Sabah*	1.8% (1.1–2.5%)	879 (546–1207)		98 (61–134)	104 (65–143)
*Farwaniya*	1.8% (1.1–2.5%)	1471 (913–2023)		163 (101–225)	175 (109–239)
*Jahra*	1.5% (0.9–2.0%)	942 (584–1296)		105 (65–144)	138 (86–190)
*Mubarak Al-Kabir*	2.0% (1.2–2.7%)	1212 (753–1665)		135 (84–185)	131 (82–180)

In the second scenario, we modeled a hypothetical 10 µg/m³ reduction in daily PM_2.5_ concentrations (Scenario 2). The attributable fractions declined to a range of 2.4% to 2.9% across hospitals. In total, this intervention would prevent 403 (95% CI: 252–551) admissions/year. For example, in Al-Adan, annual attributable admissions would drop from 306 (status quo) to 224 (95% CI: 139–308), potentially averting approximately 82 (95% CI: 51–111) cases per year.

The third scenario simulated a more ambitious 20 µg/m³ reduction in PM_2.5_ levels (Scenario 3). Here, the attributable fractions dropped further, ranging from 1.5% in Jahra to 2.0% in Mubarak Al-Kabir. The estimated annual reductions in hospital admissions were even more pronounced reaching up to 810 (95% CI: 505–1109) admissions/year.

## Discussion

This study provides the first granular evidence in Kuwait and the region linking fine particulate pollution to burdens at every public hospital in the country. We found that approximately 3–4% of cardiopulmonary hospital admissions were attributable to PM_2.5_ exposure in different hospital service areas. Across the six major public hospitals in Kuwait, this proportion corresponded to roughly 1,500 additional cardiopulmonary admissions per year that are linked solely to PM_2.5_. In simulated hypothetical scenarios, we found that even modest reductions in fine particulate pollution can yield measurable and meaningful reductions in hospital admissions burden. The gains are consistent across all hospitals. Importantly, the results highlight Farwaniya, Al-Adan, and Mubarak Al-Kabir as the highest-burden service areas; potentially making them priority targets for mitigation strategies.

Our analysis considered overall PM_2.5_ mass regardless of source. Kuwait’s pollution mix includes both natural dust and human-related emissions [17]. Source apportionment studies showed that roughly half of Kuwait’s fine particulate matter comes from wind-blown desert dust, while the remainder is attributable to anthropogenic sources such as oil combustion, industrial activities, and traffic [18–20]. This means a large portion of the PM_2.5_ burden is theoretically preventable. Regional findings in the Middle East is also pointing that the fraction of particulate pollution is largely anthropogenic and therefore an obvious target for intervention [21]. Strengthening controls on man-made emissions (e.g. energy, transport) could significantly reduce population exposure even if natural dust events continue [22]; nonetheless, regional country cooperation is underway to combat natural desert dust [23].

Notably, we estimated that each 10 µg/m³ increase in daily PM_2.5_ was associated with approximately a 1% rise in cardiopulmonary hospital admissions. This concentration-response gradient aligns with prior studies from other regions, which have generally reported on the order of a 0.5–1.5% increase in cardiovascular or respiratory hospitalizations per 10 µg/m³ of PM2.5 [4]. Biologically, such associations are plausible: inhaled fine particles deposit deep in the lungs and can trigger both localized and systemic effects. Particulate matter induces pulmonary inflammation and oxidative stress, which can ‘spill over’ into systemic circulation and disrupt cardiovascular function. Particles or soluble components translocate into the bloodstream, contributing to endothelial dysfunction, pro-thrombotic changes, and autonomic nervous system imbalance [24]. Through these pathways, PM_2.5_ exposure exacerbates respiratory conditions (e.g. by provoking bronchial inflammation in asthma [25] or COPD [26]) and can acutely increase the risk of cardiac events such as myocardial infarction [27], arrhythmias [28], or stroke [29] in vulnerable individuals.

Evidence suggests that Kuwait alone cannot eliminate its anthropogenic PM_2.5_ emissions in isolation, especially with its small geographical size and the portion of the pollution that is transported across borders [18, 19, 23, 30]. Regional collaboration with neighboring countries will be essential. Gulf nations share the challenges of desert dust, dense urbanicity and petrochemical and industrial emissions. All countries in the region (Kuwait, Iraq, Iran, Saudi Arabia, Bahrain, Qatar, United Arab Emirates, Oman) experience elevated PM_2.5_ levels that far exceed global guidelines [21]. Yet, we found that very few countries in the region have published detailed analyses of air pollution’s health impacts at the local level. In a recent WHO review of 66 time-series studies on particulate matter and health worldwide, not a single study was from an Eastern Mediterranean country except for Kuwait [31]. This highlights a critical knowledge gap across the Middle East. Kuwait air pollution studies alone cannot quantify the overall public health burdens and benefits of pollution control in the entire region. That said, Kuwait’s results show that even modest PM_2.5_ reductions (e.g. 10 µg/m³) could prevent dozens to hundreds of hospitalizations per year in a country of this size. This should encourage similar analyses in neighboring countries with a much larger population size. Multi-location research initiatives and data sharing among the Middle Eastern countries would improve the evidence base and build consensus for action [32].

Our study has several limitations. First, we relied on modeled PM_2.5_ exposure data rather than direct ground measurements. The high-resolution satellite-driven model we used has good predictive performance (R² ≈ 0.71) and ensured no missing days of data, which is a major strength in an area with sparse monitoring [13]. However, modeled concentrations inherently carry some measurement error. Second, our analysis assumed a uniform exposure across each hospital service area population. Due to data constraints, we could not account for individual patients’ exact locations or indoor/outdoor activity patterns. In reality, exposure can vary within a service area, and individuals might experience different pollution levels based on micro-environments. While this could lead to exposure error, our population-weighted averaging approach aimed to capture the overall exposure in each region. Third, we focused on acute short-term effects on hospital admissions; longer-term chronic health effects (such as the development of disease or mortality) are considered the gold-standard for cost-benefit analyses [33]. Our future research will examine chronic exposure impacts. Fourth, our attributable fraction calculations used a counterfactual of 0 µg/m³ (no pollution) to estimate the maximum preventable admissions. This is a theoretical scenario. In practice, PM2.5 cannot be driven to zero, especially not the natural dust component. The suggested 10 and 20 µg/m³ reduction scenarios offer realistic policy goals for policy decisions, albeit there appears to be no PM_2.5_ safe levels [34].

## Conclusion

Many GCC countries face similar environmental challenges, with arid climates, high population densities in urban centers, and heavy dependence on fossil fuel-based industries contributing to elevated particulate matter levels. Moreover, natural dust sources contribute to the high particulate pollution in the region. Yet, local epidemiological evidence remains limited, hindering the adoption of more protective air quality standards. Kuwait’s experience now demonstrate that modest hypothetical pollution reduction scenarios could significantly lower the healthcare burden, particularly in areas with the highest hospital admissions.

## Data Availability

Daily temperature data were obtained from the ERA5-Land daily aggregated dataset provided by the Copernicus Climate Data Store (CDS) and are publicly available through the CDS platform. The high-resolution PM₂.₅ prediction data used in this study were generated using previously published models described in Li et al. (2021) and Li et al. (2022). The underlying prediction datasets are available from the model authors upon reasonable request. Hospital admissions data were obtained from the Kuwait Ministry of Health and contain confidential patient-level information. These data are not publicly available due to privacy regulations. Researchers interested in accessing the health data may contact the Kuwait Ministry of Health, subject to appropriate ethical approvals and data-sharing agreements.
